# Effects of oral isotretinoin therapy on the nasal cavities^☆^^☆☆^

**DOI:** 10.1016/j.bjorl.2018.10.004

**Published:** 2018-11-12

**Authors:** Hamdi Tasli, Aslan Yurekli, Mert Cemal Gokgoz, Omer Karakoc

**Affiliations:** a Birecik State Hospital, Department of Otolaryngology, Head and Neck Surgery, Sanliurfa, Turkey; b Bayburt State Hospital, Department of Dermatology, Bayburt, Turkey; c Siirt State Hospital, Department of Otolaryngology, Head and Neck Surgery, Siirt, Turkey; d Gulhane Medical School, Department of Otolaryngology, Head and Neck Surgery, Ankara, Turkey

**Keywords:** Isotretinoin, Rhinomanometry, Acne, Saccharine test, Isotretinoína, Rinomanometria, Acne, Teste de sacarina

## Abstract

**Introduction:**

Isotretinoin (13 cis-retinoic acid) is the most effective treatment for acne vulgaris and is the only treatment option that can provide either remission or a permanent cure.

**Objective:**

The aim of this study was to use both subjective and objective methods to assess the nasal complaints of patients with severe acne who received oral isotretinoin therapy.

**Methods:**

Fifty-four subjects were enrolled in the study. All the subjects were assessed with subjective (NOSE and VAS questionnaires) and objective (rhinomanometry and saccharine) tests to determine the severity of their nasal complaints.

**Results:**

The mean severity scores (min: 0; max: 100) for nasal dryness/crusting and epistaxis were 0.47 ± 1.48 (0–5); 0.35 ± 1.30 (0–5) at admission, 3.57 ± 4.45 (0–10); 2.26 ± 4.71 (0–20) at the first month, and 4.28 ± 6 (0–20); 2.26 ± 4.71 (0–20) at the third month of the treatment respectively. Total nasal resistance of 0.195 ± 0.079 (0.12–0.56) Pa/cm^3^/s at admission, 0.21 ± 0.084 (0.12–0.54) Pa/cm^3^/s at the first month, and 0.216 ± 0.081 (0.14–0.54) Pa/cm^3^/s at the third month.

**Conclusion:**

Oral isotretinoin therapy can cause the complaint of nasal obstruction. In addition, nasal complaints, such as dryness/crusting and epistaxis, significantly increase in patients during the therapy schedule.

## Resumo

**Introdução:**

A isotretinoína (ácido-13 cis-retinóico) é o tratamento por via oral mais eficaz para acne vulgar e é a única opção de tratamento que pode produzir remissão ou cura permanente.

**Objetivo:**

Usar métodos subjetivos e objetivos para avaliar as queixas nasais de pacientes com acne grave que receberam terapia com isotretinoína oral.

**Método:**

Foram incluídos no estudo 54 indivíduos. Todos os indivíduos foram avaliados por meio de testes subjetivos (questionários NOSE e escala EVA) e objetivos (rinomanometria e teste de sacarina) para determinar a gravidade de suas queixas nasais.

**Resultados:**

Os escores médios de gravidade (min: 0; max: 100) para ressecamento/crostas e epistaxe nasal foram de 0,47 ± 1,48 (0-5); 0,35 ± 1,30 (0-5) no início, 3,57 ± 4,45 (0-10); 2,26 ± 4,71 (0-20) no primeiro mês e 4,28 ± 6 (0-20); 2,26 ± 4,71 (0-20) no terceiro mês do tratamento, respectivamente. A resistência nasal total foi de 0,195 ± 0,079 (0,12 a 0,56) Pa/cm^3^/s no início, 0,21 ± 0,084 (0,12 a 0,54) Pa/cm^3^/s no primeiro mês e 0,216 ± 0,081 (0,14 a 0,54) Pa/cm^3^/s no terceiro mês.

**Conclusão:**

A terapia com isotretinoína por via oral pode resultar em queixa de obstrução nasal. Além disso, queixas nasais tais como ressecamento/formação de crostas e epistaxe, aumentam significativamente nos pacientes durante o esquema terapêutico.

## Introduction

Acne is a chronic dermatologic disease characterized by lesions and scarring that last a lifetime.^1^ This disorder is more common and more severe in adolescence (70%–87% of the adolescent population) and its incidence gradually decreases in the adult years.^2^ It has a complex pathophysiology, involving abnormal keratinization, hormonal dysfunction, bacterial growth, and immune hypersensitivity.^3^ It can be divided traditionally into three categories according to the lesion type and the symptom presentations: mild, moderate, and severe. The most widely accepted form of treatment for severe acne is oral isotretinoin (13 cis-retinoic acid).

Oral isotretinoin was first approved for the management of acne vulgaris in 1982.^4^ It was originally indicated for treatment at a dose of 1–2 mg/kg/day to a cumulative dose of 120–150 mg/kg, usually administered over 4–5 months.^5^ Although it has a favorable safety profile, the major limitation of isotretinoin is its well-described adverse effect. The most common adverse effect is mucocutaneous dryness of the epidermal surfaces, giving rise to cheilitis, xerosis, and dermatitis.^6^ Despite the wide range of adverse effects including nasal complaints as epistaxis, none of the studies have mentioned the feeling of nasal obstruction and dryness/crusting.

The aim of this study was to use both subjective and objective methods to assess the nasal complaints of patients with severe acne who received oral isotretinoin therapy.

## Methods

Patients with severe acne who were treated with oral isotretinoin were enrolled in this study between August 10, 2014 and May 10, 2016. The study was carried out according to the Declaration of Helsinki and had been previously approved by the local review board (2015–269). All data were collected prospectively, and each subject was enrolled in the study after providing written informed consent. All the subjects filled out the NOSE and VAS questionnaires to determine the severity of their nasal complaints. Two objective methods – anterior rhinomanometry and saccharine tests – were also performed for each patient. These questionnaires and tests were conducted for all patients at admission and in the first and third months of the oral isotretinoin therapy schedule, and the results were compared statistically to reveal changes in the nasal complaints during the treatment process. The cumulative treatment dose, which was more reproducible and reliable than the daily dose, was used as the measurement in the present study. The isotretinoin treatment was administered at a cumulative dose of 120 mg/kg for 6 months.

### Subjective assessment of the nasal airway

The NOSE scale consists of five obstruction-related items (nasal congestion or stuffiness, nasal blockage or obstruction, trouble breathing through the nose, trouble sleeping, inability to get air through the nose during exercise) and is an easy method for determination of the severity of the complaints. Two questions regarding the severity of nasal dryness/crusting and epistaxis were also asked and scored in addition to the NOSE scale. All items were scored using a 5 point Likert scale and scaled to a total score of 0–100. Higher scores indicated greater nasal obstruction and more severe symptoms of nasal dryness/crusting and epistaxis. The VAS allows patients to rate their symptoms on a 10 cm linear scale, where 0 corresponds to symptoms that are not troublesome at all and 10 is the most troublesome symptoms imaginable.^7^

### Objective assessment of the nasal airway

Anterior rhinomanometry was used for objective evaluation of nasal obstruction. During the test, the patient was instructed to sit in an upright position and to breathe quietly for 20–30 min with a minimum air flow of 300 cm^3^/s. With this method, nasal airflow was measured from one nostril at a time and the pressure-sensing tube was swapped from one side to the other. Therefore, the pressure/flow curves and nasal resistance or conductance measurements were determined separately for each nasal passage, and the total was then calculated.^8^

The saccharine test was used to determine mucociliary clearance (MCC). Each subject was seated and positioned with the head slightly extended. A saccharine granule was 2 to 3 mm in diameter and placed by the tester, under visual control, 2 cm inside the right nostril. Each subject was instructed to swallow every 30 seconds, determined with a chronometer. The subject used a stopwatch to indicate the time for the first perception of the sweet taste of the saccharine and recorded the time in minutes. Normal nasal MCC should range between 9 and 17 min.^9^

### Inclusion/exclusion criteria

Only adult patients with severe acne were recruited. After taking a detailed medical history, a complete otorhinolaryngologic examination was performed, including nasal endoscopy after decongestion. The exclusion criteria included age below 18 years, any medical therapy within 6 months, chronic rhinosinusitis according to EPOS criteria,^10^ inflammatory or infectious sinus disease, allergic rhinitis, head and neck radiotherapy history, sino-nasal malignancy, history or clinical evidence of any nasal surgery.

### Statistical analysis

The responsiveness of the questionnaire was assessed by comparing the NOSE scores at admission and during a 3 month oral isotretinoin treatment. The Wilcoxon signed-rank test was applied to measure the magnitude of the effect for the statistical evaluation and to compare the subjective and objective test results. Statistical analyses were performed using the Statistical Package for Social Sciences software (SPSS 17.0 for Windows; SPSS Inc., IL, USA). Values of *p* < 0.05 were considered statistically significant.

## Results

Fifty-four subjects were enrolled in the study. The mean age was 21 ± 3 (18–33 years). The treatment with oral isotretinoin was stopped because of adverse side effects in 12 of the 54 patients. Three patients could not tolerate the objective tests, but they performed the subjective tests and seven patients did not attend regular follow-up, but we questioned these patients by phone and received their subjective responses. We completed the study with subjective tests administered to 42 of 54 patients and objective tests administered to 32 of 54 patients at admission and at the first and third months of the oral isotretinoin therapy schedule.

### Subjective tests

The mean NOSE scores were 14.04 ± 16.49 (0–50) at admission, 20.11 ± 20.34 (0–75) at the first month, and 19.04 ± 19.63 (0–75) at the third month of the treatment. The mean VAS scores were 1.59 ± 1.43 (1–3) at first admission, 2.14 ± 1.8 (2–3) at the first month, and 2.21 ± 1.91 (2–3) at the third month of the treatment (Table 1). Only 4 (9.5%) of the 42 patients had complaints of dryness/crusting at admission. At the first and third months, 18 (43%) patients had this complaint. The mean severity scores (min: 0; max: 100) for nasal dryness/crusting were 0.47 ± 1.48 (0–5) at admission, 3.57 ± 4.45 (0–10) at the first month, and 4.28 ± 6 (0–20) at the third month of the treatment. Only 3 (7%) of the 42 patients described a complaint of epistaxis at admission. At the first and third months, 13 (31%) and 11 (26.2%) patients described this complaint. The mean severity scores of epistaxis (min: 0; max: 100) were 0.35 ± 1.30 (0–5) at admission, 2.61 ± 4.45 (0–15) at the first month, and 2.26 ± 4.71 (0–20) at the third month of the treatment (Table 2).

**Table 1:** Subjective and objective test results of patients with severe acne who received oral isotretinoin therapy.

	Subjective test				Objective tests					
	NOSE scale		VAS scale		Rhinomanometry				Saccharine test (min)	
	Score ± SD	*p*	Score ± SD	*p*	Nasal resistance (Pa/cm^3^/s)		Nasal flow (cm^3^/s)		Score ± SD	*p*
					Score ± SD	*p*	Score ± SD	*p*		
Admission	14.04 ± 16.49	<0.05	1.59 ± 1.43	<0.05	0.195 ± 0.079	0.26	834 ± 200	0.54	9.03 ± 2.11	<0.05
1 month	20.11 ± 20.34	0.7	2.14 ± 1.8	0.8	0.21 ± 0.084	1	803 ± 188	1	9.9 ± 2.06	0.1
3 month	19.04 ± 19.63		2.21 ± 1.91		0.216 ± 0.081		773 ± 203		9.68 ± 1.94	

SD, standard deviation.

**Table 2:** Severity scores of nasal dryness/crusting and epistaxis.

	Admission				1 month			3 month			*p*
	*n*	%	MS ± SD	*p*	*n*	%	MS ± SD	*n*	%	MS ± SD	
Dryness/crusting	4/42	10	0.47 ± 1.48	<0.05	18/42	43	3.57 ± 4.45	18/42	43	4.28 ± 6	0.8
Epistaxis	3/42	7	0.35 ± 1.30	<0.05	13/42	31	2.61 ± 4.45	11/42	26	2.26 ± 4.71	1

MS, mean score; SD, standard deviation.

The differences between scores at admission and during therapy were statistically significant (*p* < 0.05), but the first and the third months were not statistically significant either for NOSE and VAS scales, severity of nasal dryness/crusting or for epistaxis (*p* = 0.7; 0.8; 0.8; 1).

### Objective tests

The mean score for the total nasal resistance was 0.195 ± 0.079 (0.12–0.56) Pa/cm^3^/s at admission, 0.21 ± 0.084 (0.12–0.54) Pa/cm^3^/s at the first month, and 0.216 ± 0.081 (0.14–0.54) Pa/cm^3^/s at the third month. The mean score of total nasal flow was 834 ± 200 (247–1256) cm^3^/s at admission, 803 ± 188 (383–1211) cm^3^/s at the first month, and 773 ± 203 (322–1201) cm^3^/s at the third month of therapy. The differences between the mean scores at admission and at the first month; and the first and the third months were not statistically significant for either total nasal resistance (*p* = 0.26–1) or nasal flow (*p* = 0.54–1) (Table 1).

The timing of nasal mucociliary activity, determined by the saccharine test, was 9.03 ± 2.11 min (5–15) at admission, 9.9 ± 2.06 min (7–15) at the first month, and 9.68 ± 1.94 min (6–14) at the third month (Table 1). The differences between the scores at admission and during therapy were statistically significant (*p* < 0.05), but the difference between the first and the third months was not statistically significant (*p* = 0.1).

## Discussion

Isotretinoin is the most effective treatment for acne vulgaris and is the only treatment option that can provide either remission or a permanent cure.^11^ The most common adverse effect is mucocutaneous dryness of the epidermal surfaces associated with cheilitis, xerosis, and dermatitis.^6^ In addition to these side effects, nasal complaints, such as the feeling of nasal obstruction and dryness/crusting, were evaluated in this study as these had not been examined previously. According to this study, it is revealed that oral isotretinoin therapy caused the feeling of nasal obstruction, epistaxis and dryness/crusting during the three month follow-up.

The mechanism of the nasal complaints can be explained by the histopathological changes that occur in response to oral isotretinoin treatment. Isotretinoin is a vitamin A derivative that decreases the proliferation, differentiation, and activity of sebaceous cysts by arresting their cell cycle.^11^ The secretions of the sebaceous cysts moisturize the nasal passage and determine the viscosity and elasticity of the mucus that lies atop the cilia layer of the mucosa. They protect the mucosa from dryness and provide an appropriate nasal physiology. Isotretinoin also inhibits sebaceous lipid synthesis and reduces the sebum excretion rate.^12^ These mechanisms block the development of acne, but they also cause mucocutaneous dryness, one of the common adverse effects already mentioned.^6^ The mucocutaneous dryness could lead to crusting, which could easily obstruct the nasal passage and create the complaint of nasal obstruction.

Several previous studies have evaluated epistaxis due to isotretinoin treatment. Blasiak et al. found epistaxis in 37.9% of the 116 patients with acne who used oral isotretinoin for 12 months and described epistaxis as one of the most common symptoms during the treatment.^13^ Ertam et al.,^14^ Gorpelioglu et al.,^15^ and Alzoubi et al.^16^ also found epistaxis in 23.1%, 40%, and 55.4%, respectively, of their patient study cohorts, while we noted it in 19% of our 42 patients. Epistaxis is a commonly accepted side effect of isotretinoin treatment, and it was blamed on the mucocutaneous dryness observed in all these studies; however, the mechanism leading to epistaxis still remains unclear. The mucocutaneous dryness and resulting crusting, which was seen in 33% of our patients, could damage the nasal mucosa with a traumatic effect, and this was likely the cause of epistaxis. In any case, the mucocutaneous dryness was followed by a vicious cycle of crusting and epistaxis during the therapy.

The mucociliary clearance (MCC) ensures the removal of foreign particles, pathogens, and toxins from the nasal passage, and it is a good indicator of a normal nasal physiology. Many recent studies have confirmed that the MCC is easily disturbed by toxins, drugs, smoking, sinonasal pathologies, and surgery,^9^ as well as by isotretinoin treatment.^15^ Gorpelioglu et al. evaluated 40 acne patients with the saccharine test and found that the saccharine time (ST) was prolonged during an isotretinoin therapy applied for 3 months as in this study. It is concluded that the mucocutaneous dryness due to isotretinoin treatment may increase the mucus viscosity in the nasal passage by altering the water and electrolyte balance, and they blamed these changes for the pathogenesis.^15^

Unlike the results of subjective tests, the rhinomanometry measurements revealed that there was no difference between the mean scores at admission and at the first month; and the first and the third months for either total nasal resistance and flow. This controversy can be explained by the limited number of patients. Although the differences between the scores were not statistically significant, the oral isotretinoin treatment clearly had adverse clinical effects in the patients proven with the subjective tests and the prolonged mucociliary activity could also contribute to this result.

The major limitations of this study were the limited number of patients and the short follow-up period. Twelve of the initial 54 patients enrolled in the study were excluded because of adverse side effects. The objective tests also require a substantial effort on the part of both the patient and the physician, and 7 patients failed to attend their regular follow-ups. Our findings suggest a need for further studies aimed at revealing the long-term nasal side effects due to oral isotretinoin therapy for acne treatment and the reversibility of these nasal complaints.

## Conclusion

Oral isotretinoin therapy can cause the complaint of nasal obstruction. In addition, nasal complaints, such as dryness/crusting and epistaxis, significantly increase in patients during the therapy schedule.

## Informed consent

All procedures performed in studies involving human participants were in accordance with the ethical standards of the institutional and/or national research committee and with the 1964 Helsinki declaration and its later amendments or comparable ethical standards. Informed consent was obtained from all individual participants included in the study

## Conflicts of interest

The authors declare no conflicts of interest.
